# Supplementary material for: Effects of Long-Term Blue Light Exposure on Body Fat Synthesis and Body Weight Gain in Mice and the Inhibitory Effect of Tranexamic Acid
Source: Int J Mol Sci. 2025 Jun 10;26(12):5554. doi: 10.3390/ijms26125554 (PMC12192898; doi:10.3390/ijms26125554)
Supplement: Supplementary file 1 [file ijms-26-05554-s001.zip › ijms-3628308-supplementary.pptx]

## Slide 1
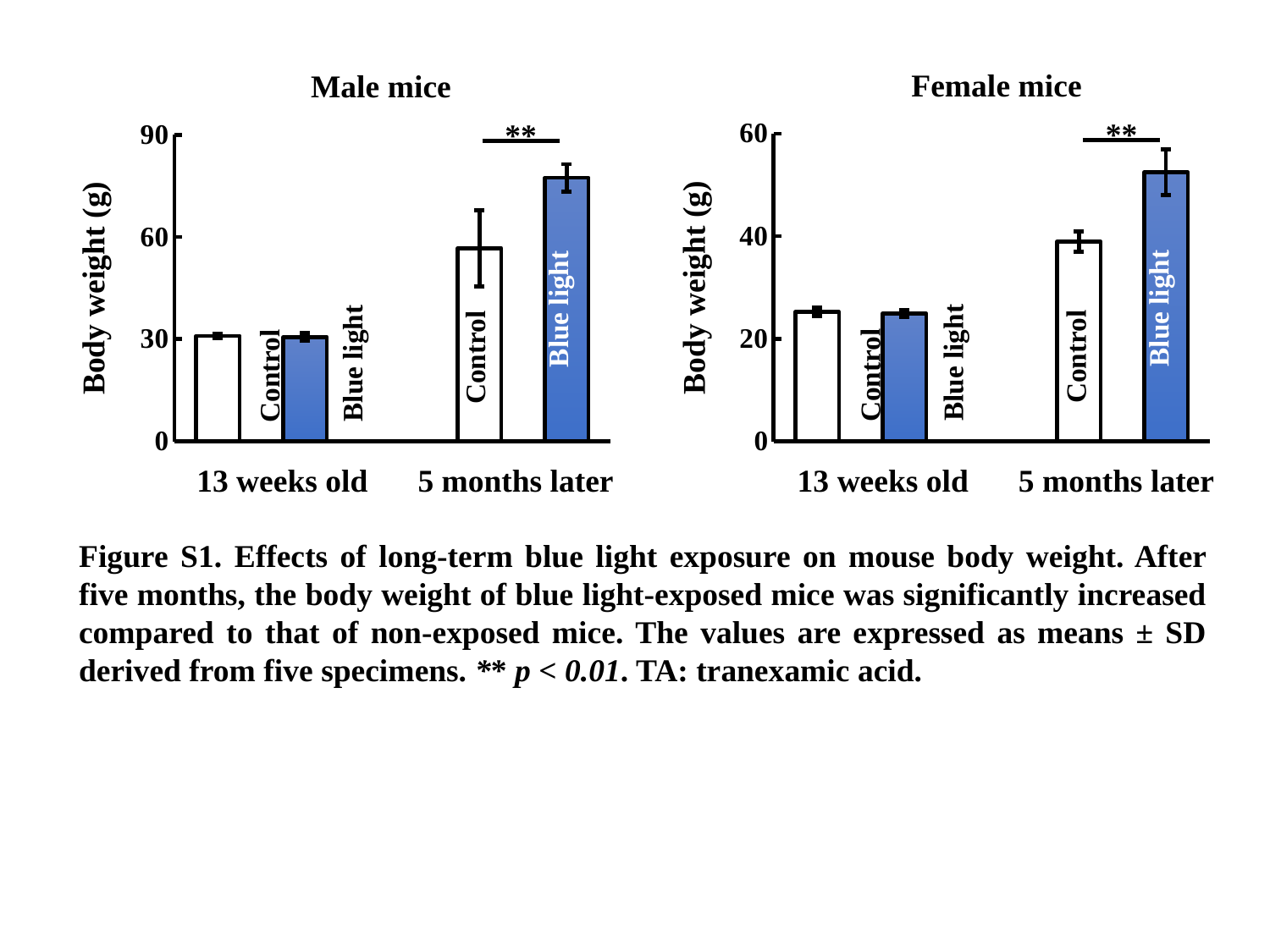

Female mice
Male mice
**
**
### Chart
| Category | |
|---|---|
### Chart
| Category | |
|---|---|Body weight (g)
Body weight (g)
Blue light
Blue light
Control
Control
Blue light
Blue light
Control
Control
13 weeks old
5 months later
13 weeks old
5 months later
Figure S1. Effects of long-term blue light exposure on mouse body weight. After five months, the body weight of blue light-exposed mice was significantly increased compared to that of non-exposed mice. The values are expressed as means ± SD derived from five specimens. ** p < 0.01. TA: tranexamic acid.

## Slide 2
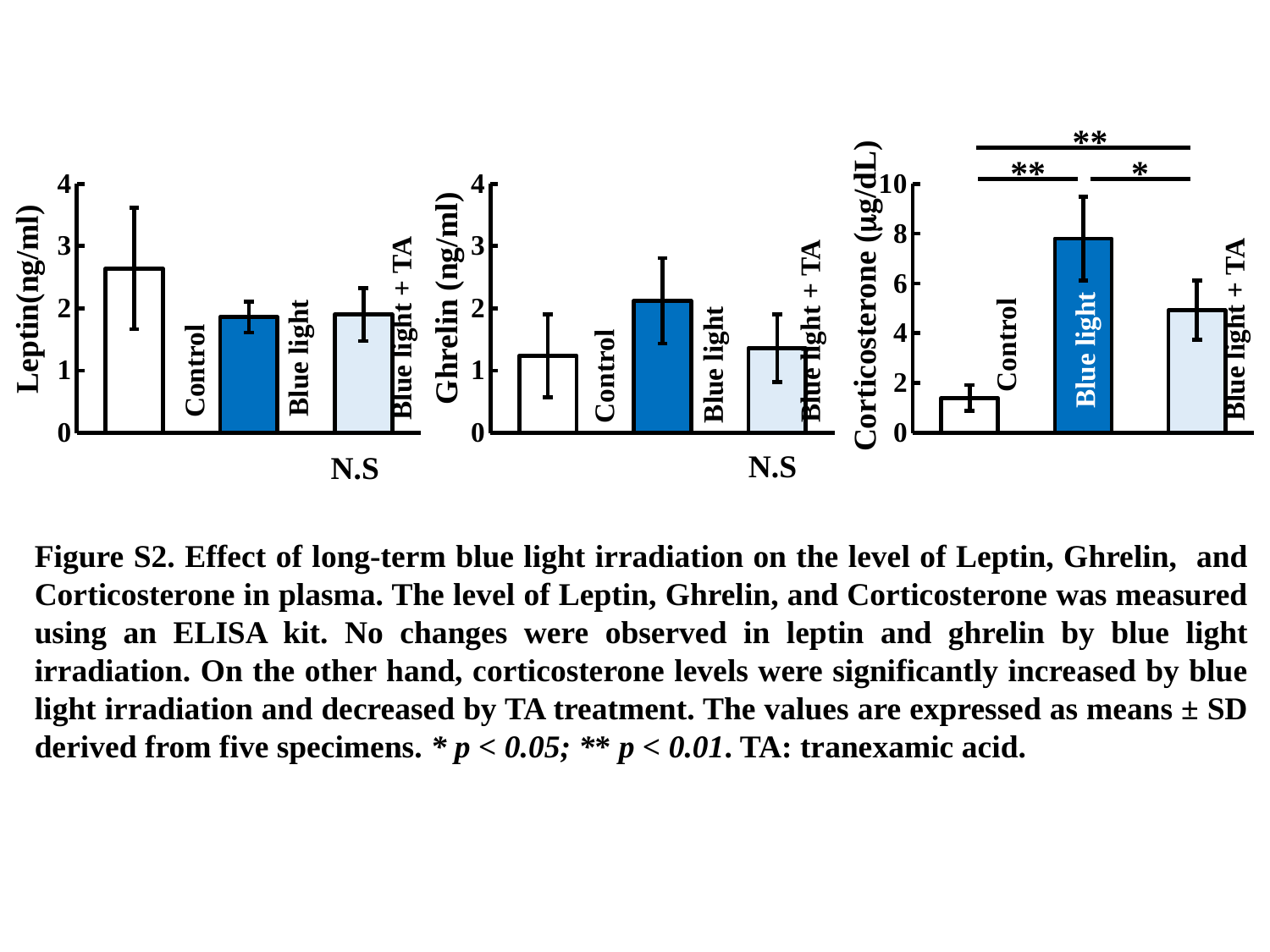

**
**
*
### Chart
| Category | |
|---|---|
### Chart
| Category | |
|---|---|
### Chart
| Category | |
|---|---|Corticosterone (mg/dL)
Ghrelin (ng/ml)
Leptin(ng/ml)
Blue light + TA
Blue light + TA
Blue light + TA
Control
Blue light
Blue light
Blue light
Control
Control
N.S
N.S
Figure S2. Effect of long-term blue light irradiation on the level of Leptin, Ghrelin, and Corticosterone in plasma. The level of Leptin, Ghrelin, and Corticosterone was measured using an ELISA kit. No changes were observed in leptin and ghrelin by blue light irradiation. On the other hand, corticosterone levels were significantly increased by blue light irradiation and decreased by TA treatment. The values are expressed as means ± SD derived from five specimens. * p < 0.05; ** p < 0.01. TA: tranexamic acid.

## Slide 3
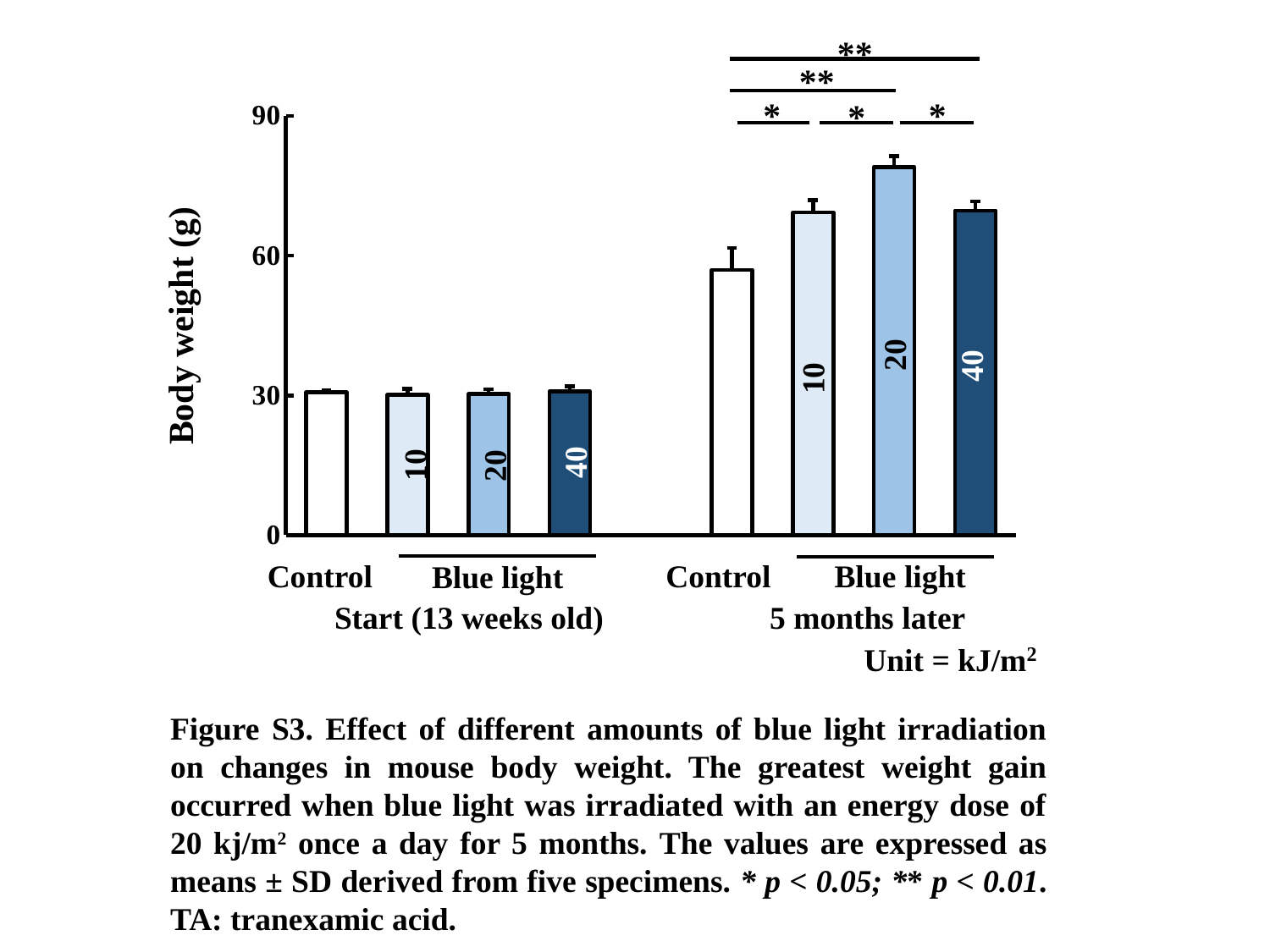

**
**
*
*
*
### Chart
| Category | |
|---|---|Body weight (g)
20
40
10
40
10
20
Control
Control
Blue light
Blue light
Start (13 weeks old)
5 months later
Unit = kJ/m2
Figure S3. Effect of different amounts of blue light irradiation on changes in mouse body weight. The greatest weight gain occurred when blue light was irradiated with an energy dose of 20 kj/m2 once a day for 5 months. The values are expressed as means ± SD derived from five specimens. * p < 0.05; ** p < 0.01. TA: tranexamic acid.

## Slide 4
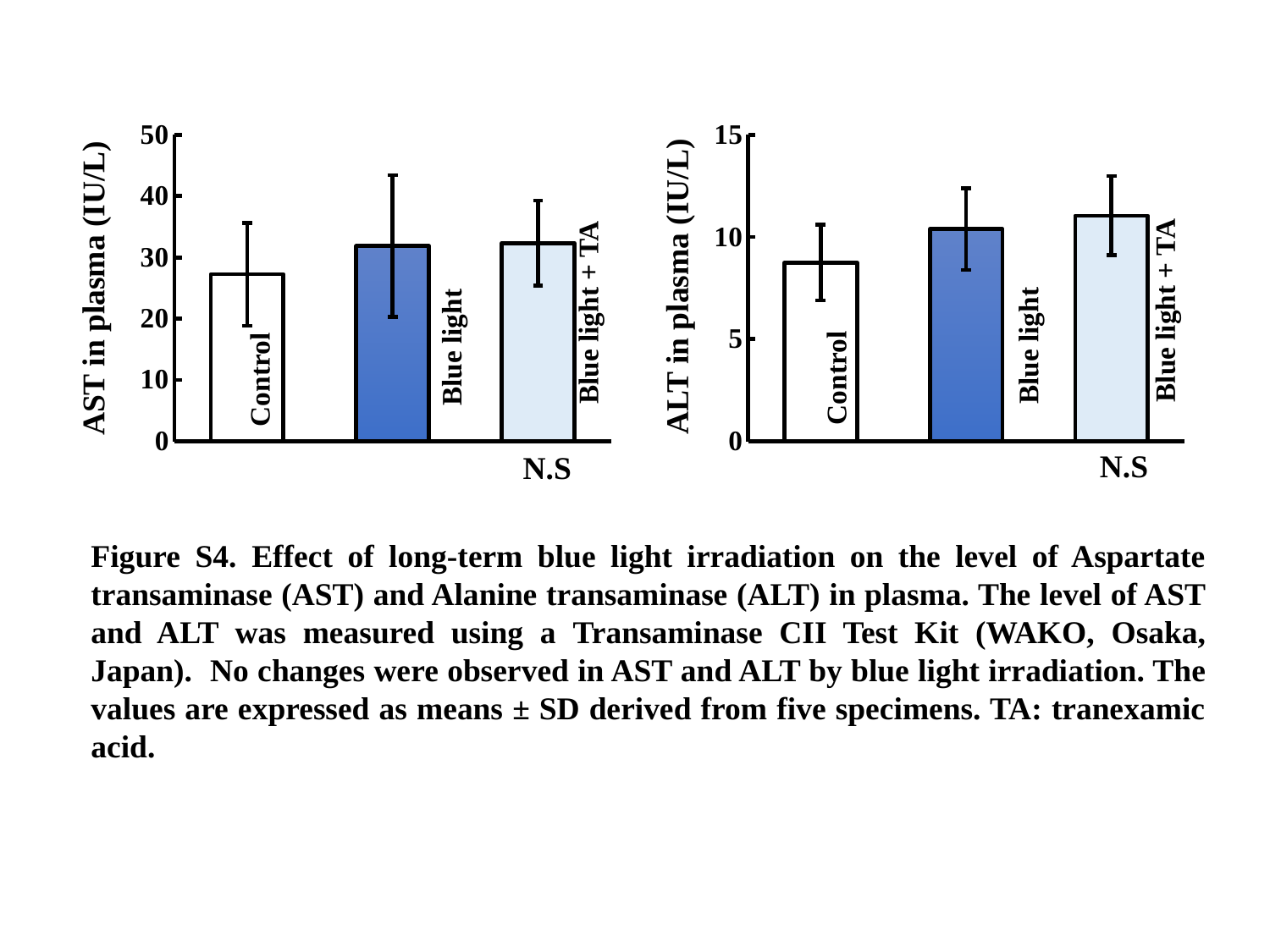

### Chart
| Category | |
|---|---|
### Chart
| Category | |
|---|---|ALT in plasma (IU/L)
AST in plasma (IU/L)
Blue light + TA
Blue light + TA
Blue light
Blue light
Control
Control
N.S
N.S
Figure S4. Effect of long-term blue light irradiation on the level of Aspartate transaminase (AST) and Alanine transaminase (ALT) in plasma. The level of AST and ALT was measured using a Transaminase CII Test Kit (WAKO, Osaka, Japan). No changes were observed in AST and ALT by blue light irradiation. The values are expressed as means ± SD derived from five specimens. TA: tranexamic acid.
